# A Comparison of Surface and Total Deltamethrin Levels of Insecticide-Treated Nets and Estimation of the Effective Insecticidal Lifetime

**DOI:** 10.4269/ajtmh.21-0144

**Published:** 2021-11-15

**Authors:** Michael Green, Mayfong Maxyay, Tiengkham Pongvongsa, Samlane Phompida, Isabel Swamidoss, Stephen Smith, Seth Irish, Paul Newton

**Affiliations:** ^1^ Division of Parasitic Diseases and Malaria, Centers for Disease Control and Prevention, Atlanta, Georgia;; ^2^ Lao-Oxford-Mahosot Hospital-Wellcome Trust Research Unit, Microbiology Laboratory, Mahosot Hospital, Vientiane, Lao PDR;; ^3^ Centre for Tropical Medicine & Global Health, Nuffield Department of Medicine, University of Oxford, Oxford, United Kingdom;; ^4^ Institute of Research and Education Development, University of Health Sciences, Ministry of Health, Vientiane, Lao PDR;; ^5^ Savannakhet Provincial Malaria Station, Savannakhet Province, Lao PDR;; ^6^ Centre of Malariology, Parasitology and Entomology, Ministry of Health, Vientiane, Lao PDR;; ^7^ U.S. President’s Malaria Initiative, U.S. Agency for International Development, Washington, District of Columbia

## Abstract

The ability to anticipate the useful lifetime of an insecticide-treated mosquito net (ITN) would provide a proactive approach for planning net distribution programs. Therefore, we used an exponential decay model of deltamethrin depletion to predict the effective insecticidal lifetime of PermaNet^®^ 2.0 nets used in the Lao PDR. Residual deltamethrin was measured using two nondestructive analytical field methods; X-ray fluorescence (total levels) and a colorimetric field test (surface levels) at 12 and 24 months postdistribution. The model assumes that the 12-month depletion rate can be used to predict future levels. The median total and surface deltamethrin levels for the Lao nets at 12 months were 31.2 and 0.0743 mg/m^2^, respectively. By defining a failed net as having total deltamethrin levels of less than 15 mg/m^2^ or a surface level less than 0.0028 mg/m^2^, it was predicted that 50% of the group of nets will fail at about 27 months after distribution.

Insecticide-treated bednets (ITNs) are recognized as important tools for reducing malaria transmission in malaria-endemic regions.^1^^–^^3^ The effectiveness of ITNs at reducing malaria transmission relies on their ability to act as chemical as well as physical barriers. Over time, the accumulation of holes, rips, and tears as well as depletion of insecticide potency, reduces the efficacy of ITNs. Although the accumulation of holes results in decreased personal protection, the presence of remaining insecticide still has the potential to reduce malaria.^4^ It has been suggested by mathematical models that 94% of transmission can be prevented if 80% of the population continues to use these nets.^4^ Thus, monitoring insecticide levels along with physical integrity are important in recognizing when an ITN is no longer effective. Although damage to the nets can be visually ascertained, monitoring insecticidal potency is often assessed by using mosquito bioassays or chemical techniques. Mosquito bioassays, such as the WHO Cone Test, are the “Gold Standard” for assessing ITNs. Although mosquito bioassays are important elements in evaluating net efficacy, it is difficult to compare net performance across geographical regions where mosquito behavior and insecticide resistance are quite variable. Therefore, this report focuses on measuring surface and total residual insecticide levels by chemical means as a practical way to monitor and predict net longevity. The chemical techniques used to measure insecticide levels usually result in the partial destruction of an ITN. Spectroscopic methods such as X-ray fluorescence (XRF)^5^^,^^6^ and surface level measurements such as the colorimetric field test for cyanopyrethroids (CFT)^7^ provide alternative insecticide analysis techniques that are nondestructive to the net, thus allowing the same net to be monitored for insecticide levels over time. The XRF method measures the total (TL) amount of insecticide per area and the CFT measures available insecticide on the net surface (SL) via an abrasion technique using filter paper. Deltamethrin adhered to the filter paper is measured using a colorimetric cyanopyrethroid analysis method.^7^ The deltamethrin molecule contains both cyano and bromine groups, thus allowing it to be detected by the CFT and XRF, respectively.^5^^,^^7^ In this report, we describe the use of both XRF and CFT methods on the same net after 12 and 24 months of use. The objective of this report is to apply an exponential decay model for predicting the effective longevity of ITNs based on TL and SL deltamethrin measured after 12 months of use. The model is based on an assumption that by 12 months, factors contributing to insecticidal loss, such as washing and storage habits have become routinely established, thereby resulting in a depletion rate constant, from which future levels can be predicted.

PermaNet 2.0^®^ mosquito nets (rebranded as Powernet [Vestergaard SA Lausanne, Switzerland]) used in the Lao PDR from 2007 to 2009^8^ were analyzed by both techniques. Details of location and CFT results have been published.^8^ The XRF analysis for TL was conducted using an Innov-X Model XT-442 analyzer (Innov-X Systems, Woburn, MA) applied directly on the net, whereas SL was measured indirectly from filter paper that had been systematically rubbed on the net.^7^ Deltamethrin levels for both XRF and CFT, expressed as mg/m^2^, are averages taken from five locations on the net as suggested by the WHO protocol for using the Cone Test.^9^ We included 37 nets that had both XRF (total) and CFT (surface) values [median (95% CI mg/m^2^)] for 12 and 24 months: 12-month XRF = 31.2 (26.6–40.5), 24-month XRF = 17.9 (15.9–20.7) and 12-month CFT = 0.0743 (0.0014–0.127), 24-month CFT = 0 (0–0.138). The deltamethrin depletion rate “b” for the first 12 months of use was determined using the following equation #1: b = ((Ln(*C*_0_) − Ln(*C*_12_))/−12 months), where *C*_12_ is the median deltamethrin concentration value for groups of nets analyzed at 12 months. If baseline levels of deltamethrin were not available, then *C*_0_ was assumed to be the manufacturer’s target dose of 55 mg/m^2^ for total levels and an equivalent surface level of 1.02 mg/m^2^. The concentration at a particular time (*C*_t_) can be estimated with a typical exponential equation #2: *C*_t_ = C_0_ × exp(b × t). If *C*_t_ is a fixed value, that is, a threshold concentration where a net is considered as “failed,” rearrangement of equation #2 yields the time it takes to reach the given particular threshold concentration [*t* = ((Ln(*C*_t_/*C*_0_))/−b]). Green et al. (2009) determined that a surface level (SL) value of 0.0028 mg/m^2^ is equivalent to the optimum concentration required to achieve 80% mosquito mortality.^7^ The SL threshold value was determined based upon the use of a susceptible strain of the African malaria vector, *Anopheles gambiae.* Since the median value for SL at 12 months (*C*_12_) was 0.074 mg/m^2^ and *C*_0_ = 1.02 mg/m^2^, the depletion rate (b) was −0.219 mg/m^2^/month. Defining the SL threshold concentration (*C*_t_) for PermaNet2.0 as 0.0028 mg/m^2^ deltamethrin for a failed net, 50% of the Lao nets were predicted to fail at 27.0 months. In comparison, Kilian et al. (2008) suggested that a value of more than 15 mg/m^2^ for PermaNet 2.0 was needed to be optimally effective against the Kisumu strain of *Anopheles* mosquitoes.^10^ Therefore, using the TL median of 31.2 mg/m^2^ as *C*_12_ (Table 1), *C*_0_ = 55 mg/m^2^ and *C*_t_ = 15 mg/m^2^, it would take 27.5 months until 50% of the Lao nets fail. For comparison, Table 1 lists TL concentrations, depletion rates and predicted levels for PermaNet 2.0 used in studies conducted in different countries. The predicted levels of residual deltamethrin correlated well with the actual levels (Pearson correlation of 0.858, *P* < 0.0001, *N* = 22). The mosquito mortality results of a WHO Cone Test conducted on 11 randomly chosen 24-month-old Lao nets were reported to be 91% (*N* = 11).^8^ The predicted and actual 24-month percent net failure predicted from 12-month total levels from the same 11 nets was 64% (7/11). Nets with average deltamethrin levels below 15 mg/m^2^ were defined as “failed.” These two proportions were not significantly different (*P* = 0.80, Χ^2^ test). Figure 1 shows the relationship between levels found at 12 months (*y* axis) and the length of time (*x* axis) it would take to reach a chosen threshold level (*z* axis). The scale at the right-hand side of the *y* axis represents surface levels equivalent to total levels using the following relationship: [Ln(Surface Level) = (0.1273 × Total Level − 7.0), *R*^2^ = 0.86, *N* = 11]. Total net deltamethrin levels were determined from complete extraction of deltamethrin from the net material followed by analysis using a modified high-performance liquid chromatographic (HPLC) technique (CIPAC/4838). The median depletion rate constant for the 10 studies shown in Table 1 is −0.035 (95% CI: −0.092 to 0.114) mg/m^2^/month. If using the threshold concentration of 15 mg/m^2^ as determined by Killian et al.,^10^ then the median time a PermaNet 2.0 net lasts is 3.1 years. This corresponds with the statement from the manufacturer, Vestergaard; “PermaNet^®^ 2.0 is designed to last at least 20 WHO standard washes and 3 years of field use; however, the lifetime largely depends on usage and local field conditions.”^11^ It is not clear what the primary factors influencing deltamethrin depletion are. In a study conducted on nets from India and Nepal, it was concluded that washing has no significant bearing on deltamethrin levels and that handling, friction, and torsion may be more of a contributor.^12^ Manipulation or handling of the net may be represented by the frequency at which the net is put up and taken down. The percentage of the Nepalese nets that were taken down, folded, and stowed each day was 15.8% in comparison to 45.1% of Indian nets.^12^ Lower manipulation of the Nepalese nets may have contributed to the longer deltamethrin half-life of 20.6 months (Table 1) relative to the half-life of 13.8 months (Table 1) for the Indian nets. Only 2.7% of nets from the Ugandan study^10^ were stored away daily, which is consistent with the long half-life of 38.6 months shown in Table 1. A survey conducted around the same time and location as our study showed that 68% of Lao nets remained hanging during the day^13^ Assuming 32% had been taken down, the half-life of 14.7 months (Table 1), is consistent with observations made from daily net takedown percentages from Nepalese, Indian, and Ugandan nets with their respective half-lives. Storing nets that are not in use lessens the opportunities for holes, rips, and tears to develop. Although the manipulations involved with handling and folding the nets for storage may contribute to a shorter insecticidal half-life, an intact net continues to retain its barrier effect against mosquitoes.

**Figure 1.**
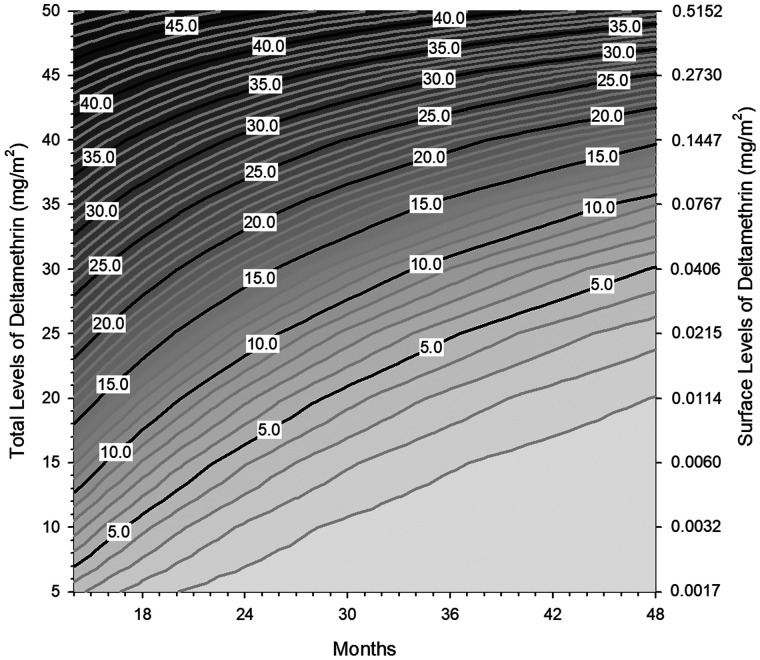
Contour plot showing the relationship between total levels of deltamethrin (*y* axis left-hand scale) and surface levels of deltamethrin (*y* axis right hand scale) with months of use (*x* axis) after a total deltamethrin threshold level (*z* axis) has been chosen.

**Table 1:** Comparison of total deltamethrin concentrations from other studies

Country	Months	Median	*N*	95% CI range	Depletion rate	Half-life	Predicted	Source
	of use	mg/m^2^			mg/m^2^/month	months	mg/m^2^	
Lao PDR	12	31.2	37	26.0–36.4	−0.047	14.7		This study
	24	17.9	37	15.5–20.3			17.7	
Kenya	12	28.8	26	19.5–38.1	−0.054	12.9		Briet et al. 2020^14^
	24	12.1	30	5.4–18.8			15.1	
	36	8.8	50	3.0–14.6			7.9	
	48	8.4	50	1.9–14.9			4.1	
Malawi	12	47.6	22	43.2–52.0	−0.012	57.6		
	24	20	25	11.1–28.9			41.2	
	36	23.7	16	−6.9–54.3			35.7	
Mozambique	12	25.5	70	21.8–29.2	−0.064	10.8		
	24	13.5	102	10.0–17.0			11.8	
	36	2.4	57	0.5–4.3			5.5	
Zambia	12	45.6	18	38.8–52.4	−0.016	44.4		Tan et al. 2016^15^
	24	19.1	18	14.2–24.0			37.8	
Uganda	0	69.2	10	59.6–78.8				Kilian et al. 2008^10^
	12	55.8	40	45.1–66.5	−0.018	38.6		
	19	44.5	40	36.3–52.7			49.2	
	25.5	32.3	38	25.3–39.3			43.8	
	37.5	28.7	40	22.1–35.3			35.3	
India	12	30.1	38	27.0–33.6	−0.050	13.8		Picado et al. 2012^12^
	24	11.6	40	9.1–14.8			20.7	
Nepal	12	36.7	25	32.1–41.9	−0.034	20.6		
	24	27.9	25	23.8–32.6			30.8	
Papua New Guinea	12	47	23		−0.013	52.9		Katusele et al. 2014^16^
	24	34	5	4.2–63.8			40.2	
	36	18	9	6.2–29.8			34.3	
	48	24	7	6.2–41.8			29.3	
	84	16	3	0.0–34.1			18.3	
Tanzania	0	58	10	55.5–60.5				Lorenz et al. 2020^17^
	10	30	48	25.1–34.9	−0.066	10.5		
	22	18.8	48	14.7–22.9			13.6	
	36	16	48	11.2–20.8			5.4	

Deltamethrin concentration value for India and Nepal are geomeans and means for Papua New Guinea and Tanzania. C_0_ = 55 mg/m^2^ for all countries except Uganda and Tanzania, where C_0_ = 69.2 mg/m^2^ and C_0_ = 58 mg/m^2^.

In conclusion, the ability to measure deltamethrin levels using two nondestructive techniques provided the opportunity to compare the surface levels with total levels of the insecticide on nets used in the Lao PDR. An exponential decay model applied to levels determined after 12 months of use gave a reasonable estimate of future levels, thus providing a means of predicting the critical time when 50% of the nets fail based on the chosen threshold concentration. The model can provide useful information for anticipating when groups of nets from a particular region lose their effectiveness.
